# The tumor microenvironment as a metabolic barrier to effector T cells and immunotherapy

**DOI:** 10.7554/eLife.55185

**Published:** 2020-05-05

**Authors:** Aaron R Lim, W Kimryn Rathmell, Jeffrey C Rathmell

**Affiliations:** 1 Department of Medicine, Division of Hematology and Oncology, Vanderbilt University Medical CenterNashvilleUnited States; 2 Vanderbilt-Ingram Cancer Center, Vanderbilt University Medical CenterNashvilleUnited States; 3 Vanderbilt Center for Immunobiology, Vanderbilt University Medical CenterNashvilleUnited States; 4 Department of Pathology, Microbiology, and Immunology, Vanderbilt University Medical CenterNashvilleUnited States; University of Paris DescartesFrance; PfizerUnited States

**Keywords:** immunometabolism, tumor microenvironment, tumor-infiltrating lymphocytes, t cells, immunotherapy, cancer

## Abstract

Breakthroughs in anti-tumor immunity have led to unprecedented advances in immunotherapy, yet it is now clear that the tumor microenvironment (TME) restrains immunity. T cells must substantially increase nutrient uptake to mount a proper immune response and failure to obtain sufficient nutrients or engage the appropriate metabolic pathways can alter or prevent effector T cell differentiation and function. The TME, however, can be metabolically hostile due to insufficient vascular exchange and cancer cell metabolism that leads to hypoxia, depletion of nutrients, and accumulation of waste products. Further, inhibitory receptors present in the TME can inhibit T cell metabolism and alter T cell signaling both directly and through release of extracellular vesicles such as exosomes. This review will discuss the metabolic changes that drive T cells into different stages of their development and how the TME imposes barriers to the metabolism and activity of tumor infiltrating lymphocytes.

## Introduction

Hanahan and Weinberg’s seminal paper ‘The Hallmarks of Cancer’ was revised in 2011 to include deregulating cellular energetics and evasion of immune destruction (Hanahan and Weinberg, 2011). Tumors fuel their rapid growth and proliferation with aerobic glycolysis, a process initially described by Otto Warburg in which cells undergo glycolysis even in the presence of oxygen (Lebelo et al., 2019). Although less energetically efficient than oxidation that occurs in most mature tissues, aerobic glycolysis shuttles intermediates into biosynthetic pathways to make amino acids, nucleotides, fatty acids and other macromolecules to support rapid anabolic growth (Pavlova and Thompson, 2016). As a consequence, glucose and amino acids can be rapidly consumed while waste products accumulate. Activated T cells also undergo a metabolic switch from oxidative metabolism to aerobic glycolysis to proliferate and develop effector function (Menk et al., 2018; Bantug et al., 2018a). Rapid proliferation and acquisition of effector function are demanding processes that require precise metabolic re-wiring. Failure of activated T cells to undergo metabolic re-wiring impairs effector function (Kouidhi et al., 2017). As T cell metabolism dictates effector function, it is now apparent that the effect of cancer cell metabolism on the tumor microenvironment (TME) may impair anti-tumor immunity, and these new hallmarks of cancer are therefore inextricably linked.

Expanded understanding of the basic biology of T cell activation has enabled immunotherapy to combat cancer, and T cell metabolism now offers the opportunity to optimize and improve these therapeutic strategies. Two of the primary immunotherapies are immune checkpoint blockade (ICB) and adoptive cell transfer (ACT). ICB is based on the use of antibodies to neutralize inhibitory immune receptors such as CTLA-4 or PD-1 to reinvigorate T cells (Baumeister et al., 2016). In contrast, ACT expands a patient’s own T cells ex vivo to direct anti-tumor immunity when transfused back into the patient. These treatment modalities have shown great promise in many types of cancer and even produce long-lasting responses in some patients (Gong et al., 2018). However, many patients fail to respond to these therapies, and metabolic barriers imposed on T cells by the TME may contribute. This review will discuss the metabolic adaptations necessary for T cells to meet changing biochemical needs throughout different stages of differentiation. We will then examine how tumor cells create a toxic milieu for T cells that enter the TME. Finally, we will provide an overview of how utilizing an understanding of T cell metabolism may inform strategies to alter the TME or enhance T cell metabolism to strengthen the immunotherapy arsenal.

### Metabolic reprogramming of T cells

**Figure 1.**
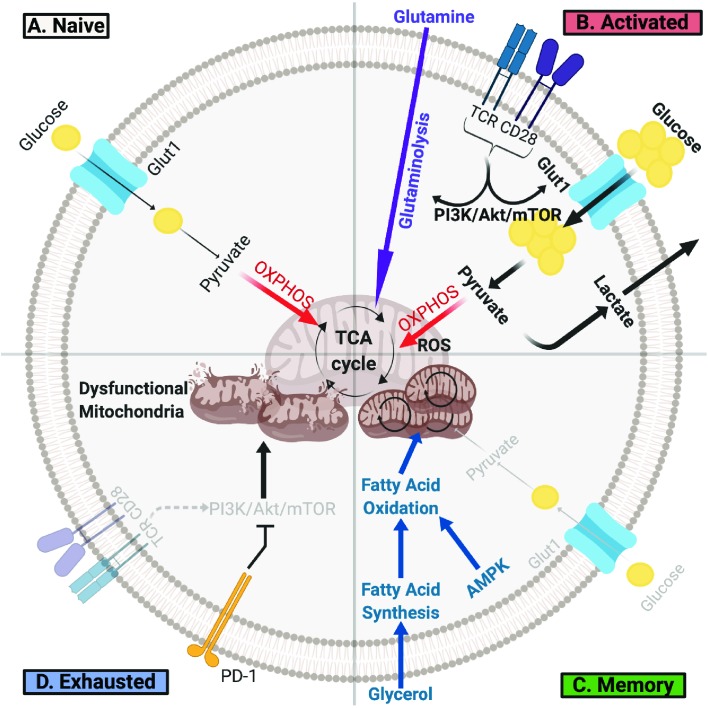
T Cells Undergo Metabolic Rewiring in Different Stages of Their Life. (**A**) Naïve T cells uptake sufficient amounts of glucose to fuel oxidative phosphorylation and survive as they survey antigens. (**B**) Upon encountering cognate antigen, activated T cells rapidly uptake glucose and glutamine to fuel their bioenergetic needs. Activated T cells perform aerobic glycolysis, which shunts products of glycolysis to biosynthetic processes necessary for proliferation and effector function and generates lactate as a byproduct. (**C**) Once the antigen is cleared, T cells can form long-lived memory cells in which AMPK signaling stimulates fatty acid oxidation. Memory T cells also increase their mitochondrial mass and spare respiratory capacity to prepare for future encounter with cognate antigen. (**D**) T cells can become exhausted if they fail to clear antigens such as during chronic infections or cancer. T lymphocytes isolated from tumors display elevated levels of PD-1, which decreases PI3K/Akt/mTOR signaling and glycolysis. Exhausted TILs rely on fatty acid oxidation, though they often have dysfunctional mitochondria and decreased mitochondrial mass as well.

There is a growing appreciation that distinct metabolic programs drive different developmental stages of a T cell throughout its lifespan [Figure 1]. After leaving the thymus, naïve T cells utilize a catabolic metabolism in which small amounts of glucose are used to generate ATP mainly through oxidative phosphorylation to support immune surveillance (Geltink et al., 2018; Chapman et al., 2020). To proliferate and gain effector function, stimulated T cells must undergo rapid metabolic reprogramming and switch to aerobic glycolysis to support anabolic metabolism and exit quiescence (Geltink et al., 2018; Chapman et al., 2020). Although fewer ATP molecules are generated per glucose molecule, aerobic glycolysis allows T cells to build substrates needed for growth and proliferation and is essential for effector differentiation (Menk et al., 2018). Metabolic reprogramming from catabolism to anabolism is initiated upon T Cell Receptor (TCR) recognition of cognate antigen presented on major histocompatibility complex (MHC) and with the help of CD28-mediated co-stimulation. TCRs cluster and signal to the phosphatidtyl-inositide-3 kinase (PI3K)/AKT/mTORC1 pathway to upregulate nutrient uptake, glycolysis and, to a lesser extent, oxidative phosphorylation (Sena et al., 2013; Frauwirth et al., 2002). T cell metabolism is further re-wired by transcription factors such as c-Myc and hypoxia inducible factors (HIFs), which transcribe genes essential for T cell activation and regulate glycolysis and glutaminolysis (Wang et al., 2011; Palazon et al., 2017). Importantly, limiting glucose availability or inhibiting glycolytic enzymes impairs effector T cell proliferation and cytokine production (Macintyre et al., 2014; Chang et al., 2013; Angiari et al., 2019). Increased amino acid uptake is also essential, and deficiency of glutamine, neutral amino, or essential amino acid transporters can impair effector T cell development (Sinclair et al., 2019; Sinclair et al., 2013; Najjar et al., 2019; Johnson et al., 2018). While glutamine uptake itself is required for T cell activation, glutamine metabolism appears to play a complex role, as glutaminolysis can suppress effector T cell differentiation and function (Johnson et al., 2018; Leone et al., 2019). In addition to these pathways, mitochondria undergo physical and functional changes required for efficient T cell activation. T cell activation with CD28 co-stimulation leads to mitochondrial fragmentation that can reduce oxidative efficiency in effector T cells (Buck et al., 2016), although CD28 co-stimulation increased respiration under glucose limiting conditions (Frauwirth et al., 2002). This distinction may be due to findings that CD28 co-stimulation can increase T cells spare respiratory capacity and remodel cristae, allowing memory T cells to manage metabolic stress and quickly to future stimuli (Klein Geltink et al., 2017). The mitochondria of T lymphocytes also undergo significant proteomic changes that favor one-carbon metabolism critical for nucleotide synthesis, methylation, and redox balance in T cell activation (Ron-Harel et al., 2016). Meanwhile, mitochondrial reactive oxygen species (ROS) production promotes nuclear factor of activated T cells (NFAT) activation and IL-2 production (Sena et al., 2013).

Although most of our understanding of T cell metabolism comes from in vitro or ex vivo studies, the question of whether this translates in vivo has been understudied. A recent study by Ma et al. compared CD8^+^ T cell metabolism in vivo versus in vitro by tracing glucose metabolism with ^13^C-glucose IV infusions in a listeria monocytogenes infection mouse model (Ma et al., 2019a). Their results showed that CD8^+^ T cells in situ had reduced lactate production and higher rates of oxidative metabolism and serine metabolism compared to in vitro CD8^+^ T cells that adopted a metabolic phenotype consistent with the Warburg effect (Ma et al., 2019a). The environment may contribute to these metabolic differences, since in vitro conditions often supply high amounts of glucose. Indeed, when the in vivo effector T cells were cultured in the same in vitro media conditions, the cells secreted more lactate (Ma et al., 2019a). These data suggest that T cells in vivo utilize glucose primarily as an anabolic source with alternate fuels such as glutamine supporting ATP, whereas glucose may play a broader anabolic and energetic role in vitro that would necessitate higher glucose flux and subsequent lactate production to maintain redox balance. Re-assessing the nutrients in vitro cultures of T cells to better reflect in vivo conditions is necessary to more precisely study T cell metabolism under a variety of contexts such as cancer.

Long-lived memory T cells that form after the induction of an immune response also have distinct metabolic features. Unlike activated T lymphocytes, memory T cells have low mTOR signaling and high AMPK signaling, promoting fatty acid oxidation (Araki et al., 2009; Pearce et al., 2009). To support this state, memory T cells increase their uptake of glycerol, which is used to synthesize fatty acids that subsequently fuel fatty acid oxidation (Cui et al., 2015). In contrast to naïve or activated T cells, memory T cells have more mitochondrial mass and mitochondrial spare respiratory capacity (van der Windt et al., 2012) and are poised to rapidly induce aerobic glycolysis (Gubser et al., 2013; Bantug et al., 2018b). This unique metabolic program allows memory T cells to quickly respond upon encountering the cognate antigen (Fraser et al., 2013). However, when effector T cells cannot effectively clear antigens such as during chronic infection or cancer, they may not form memory T cells and instead may become exhausted (McLane et al., 2019). Exhausted T cells are a distinct class of T lymphocytes characterized by lower proliferative capacity, survival, and cytokine production. They also express different transcription factors and high amounts of inhibitory receptors such as PD-1, leading to metabolic re-wiring (Khan et al., 2019). In particular, PD-1 signaling promotes the switch from glycolysis to fatty acid oxidation by suppressing AKT and mTOR activity (Saeidi et al., 2018). These functionally impaired T cells also have impaired mitochondria (Schurich et al., 2016), and improving mitochondrial function with anti-oxidants restored T cell activity (Fisicaro et al., 2017). In addition, overexpression of peroxisome proliferator-activated receptor-γ coactivator (PGC-1α) increased glucose uptake and decreased mitochondrial mass and polarization, and it enhances the function of exhausted T cells in both a lymphocytic choriomeningitis virus (LCMV) infection (Bengsch et al., 2016) and in a B16 melanoma model (Scharping et al., 2016). Overall, T cells must adapt their metabolism to fuel a program that meets their changing needs at different activation, functional, and microenvironmental stages.

### T cells encounter a hostile metabolic environment in tumors

T cells isolated from tumors often show signs of exhaustion and have distinct metabolic signatures (Scharping et al., 2016; Siska et al., 2017). For example, tumor-infiltrating lymphocytes (TILs) isolated from clear cell renal cell carcinoma patients show decreased glucose uptake as well as small, fragmented mitochondria with elevated ROS (Siska et al., 2017). Bypassing these metabolic defects by supplementing with pyruvate or adding ROS scavengers partially restored TIL activation (Siska et al., 2017). TILs in a murine melanoma model have also been shown to have dysfunctional enolase (Gemta et al., 2019) and reduced mitochondria biogenesis (Scharping et al., 2016), and tumor-associated lactate and cholesterol suppress TIL function (Brand et al., 2016; Ma et al., 2019b). In addition, TILs can infiltrate sarcoma tumors but do not produce cytokines until after checkpoint blockade (Gubin et al., 2014). These studies suggest that antigen recognition and infiltration into tumors alone are insufficient for an antitumor response, and that the tumor metabolic microenvironment can directly suppress T cells.

#### Hypoxia

T cells are primed in nutrient-rich lymphoid tissues but enter tumors where cancer cell metabolism and poor vascular exchange may lead to a fierce competition for resources. One hostile aspect of the TME that infiltrating T cells encounter is hypoxia, created by the high metabolic rate of tumor cells in conjunction with inadequate vasculature. Cancer cells can adapt to thrive under low oxygen conditions, and several studies have shown the association of hypoxia with angiogenesis, metastasis, and chemoresistance (Harris, 2002; Wilson and Hay, 2011). Under low oxygen states, the transcription factor hypoxia-inducible factor (HIF) is free from its negative regulator von Hippel-Lindau (VHL) to upregulate its target genes (Marx, 2004). Studies have begun to show how hypoxia can lead to metabolic dysfunction in T cells, though it still remains a complex subject. There are studies that suggest hypoxia can have an immunostimulatory effect on T cells in the TME. For example, lack of oxygen stabilizes HIF-1α, which increases pyruvate dehydrogenase kinase and lactate dehydrogenase A expression and thus decreases oxidative phosphorylation (Papandreou et al., 2006; Kim et al., 2006). Hypoxic CD8^+^ T cells increase granzyme B packaging into granules and reject B16 tumors in mice more efficiently than normoxic T cells (Gropper et al., 2017), and loss of HIF-1α, but not HIF-2α, inhibited the activity and migration of OT-I T cells and enhanced tumorigenesis (Palazon et al., 2017). In contrast, other studies indicate an immunosuppressive role for hypoxia. For example, HIF-1α is known to upregulate PD-L1 on myeloid-derived suppressor cells (MDSCs), which leads to T cell exhaustion, and can promote the generation of regulatory T cells (Shen et al., 2019; Noman et al., 2014; Ben-Shoshan et al., 2008). Highly oxidative cancer cells can lead to areas of hypoxia, and this has been associated with decreased T cell response to ICB (Najjar et al., 2019). Indeed, T cells have been shown to avoid areas of hypoxia in the TME, but mice breathing 60% oxygen displayed enhanced CD8 T cell infiltration into the TME and increased tumor regression and survival in multiple tumor models (Hatfield et al., 2015). These studies underscore that there is likely a fine balance with HIF-1α expression in TILs that can later their function in the TME. Further investigation is warranted to tease out the effects of hypoxia in the TME.

#### Nutrient competition and metabolic byproducts

Consistent with both cancer cells and effector T cells utilizing aerobic glycolysis in tumor microenvironments that can have poor vascular exchange, evidence supports competition for available nutrients that can impair TILs [Table 1]. Indeed, intratumor glucose levels have been measured and can be significantly reduced in some settings (Scharping et al., 2016; Ho et al., 2015), although glucose levels can remain unchanged in others (Siska et al., 2017). Cancer cells may contribute to reduced glucose availability as rapid glucose consumption by mouse sarcoma cells was found to restrict the effector function of TILs and thus permitted tumor progression (Chang et al., 2015). Tumor regression and TIL function were inversely associated with the capacity of sarcoma cells to perform aerobic glycolysis. Another study found that culturing T lymphocytes in conditioned media from ovarian cancer cells decreased expression of the methyltransferase EZH2 in T cells and their polyfunctionality, and these effects were abrogated upon glucose supplementation (Zhao et al., 2016). In addition, treating human ovarian cancer-specific T cells with an EZH2 inhibitor prior to adoptive transfer led to increased tumor growth in a humanized ovarian cancer mouse model (Zhao et al., 2016). These data suggest that glucose metabolism can regulate T cell polyfunctionality by modulating EZH2 expression, although whether one should induce EZH2 expression in cancer patients is complicated since some cancers acquire gain-of-function mutations in this methyltransferase (Kim and Roberts, 2016). It would be interesting to test whether inducing EZH2 expression in adoptively transferred T cells could overcome the effects of glucose deprivation in the TME. Insufficient glucose in tumors may also impair T cell signaling to restrain anti-tumor immunity through a phosphoenolpyruvate-dependent regulation of calcium signaling (Ho et al., 2015). Collectively, these studies highlight the importance of glucose in the TME for T cell function.

**Table 1.** Hostile Conditions in The Tumor Microenvironment Impair T Cell Metabolism and Anti-Tumor Immunity. Cancer cell metabolism, improper blood vessel formation, and extracellular vesicles all contribute to a toxic milieu deficient in key nutrients, such as glucose and oxygen, and high in waste products, such as lactate. Consequently, TILs entering the TME are deprived of key nutrients, disturbing metabolic processes critical for their anti-tumor functions.

Component of the TME	Impact on T Cell Metabolism	Effect on Anti-Tumor Immunity
Hypoxia	• Stabilizes HIF-1α • Increases pyruvate dehydrogenase kinase, blocking the conversion of pyruvate to acetyl-CoA and thus mitochondrial respiration and ROS production • Increases lactate dehydrogenase A expression and inactivates pyruvate dehydrogenase, shunting pyruvate to lactate	• Increases granzyme B packaging into granules, leading to rejection of B16 tumors in mice • Upregulates PD-L1 expression on MDSCs • Decreases T cell infiltration
Depletion of Glucose	• Reduces aerobic glycolysis • Decreases levels of phosphoenolpyruvate, which regulates calcium and NFAT signaling	• Suppresses TIL effector function • Reduces EZH2 expression, decreasing T cell polyfunctionality
Accumulation of Lactate	• Impedes lactic acid export from CD8^+^ T cells, which slows down glycolysis and reduces ATP levels • Decreases NFAT levels and translocation to the nucleus	• Inhibits T cell proliferation, activation, and function • Induces T and NK cell apoptosis
Tumor-derivedExtracellular Vesicles (EVs)	• Modulates the metabolism of tumor associated macrophages and other cell types. • Effects of EVs on T cell metabolism are currently unknown	• Suppresses TIL anti-tumor function. • However, blocking EV biogenesis induces T cell activation, proliferation, and effector function.

In addition to potential limitations to available glucose, other nutrients may also become limiting in context specific manners. A mass spectrometry-based analyses of institutional fluid from a pancreatic ductal carcinoma mouse model showed depletion of some essential and branch chain amino acids, while others were enriched in the tumor microenvironment (Sullivan et al., 2019). Importantly, the tumor location, diet, and cancer type could shift the metabolic composition of the tumor interstitial fluid, indicating that the overall tumor context may exert a strong influence over the TME. Strategies to increase alternative programs, such as lipid metabolism driven by PGC1α (Scharping et al., 2016) or PPARα (Zhang et al., 2017), may rewire metabolism and overcome these metabolic deficiencies, although the metabolic implications of these adaptations for T cell proliferation and effector function remain poorly understood.

Tumor cells also produce byproducts detrimental to T cells. Indoleamine 2,3-dioxygenase (IDO) in cancer cells catalyzes oxidative catabolism of tryptophan, thus dampening antitumor immune responses by depleting this essential amino acid and producing kyneurenine, which generates immunosuppressive regulatory T cells (Tregs) (Munn and Mellor, 2013; Mezrich et al., 2010). These Tregs in turn can promote IDO expression on dendritic cells, further increasing kynurenine and depleting tryptophan in the TME (Fallarino et al., 2003). Another toxic byproduct produced by cancer cells is lactate (Siska and Rathmell, 2015). High lactate concentrations produced by tumor cells impeded lactic acid export in CD8^+^ T cells and thus suppressed their effector function (Fischer et al., 2007). Tumor-associated expression of lactate dehydrogenase A is associated with lower survival and impaired T cell activity in melanoma patients (Brand et al., 2016). In contrast, a recent proteomic analysis of melanoma showed that tumors with higher oxidative phosphorylation and lipid metabolism had increased antigen presentation and were associated with response to anti-PD-1 or TIL-based immunotherapy (Harel et al., 2019). One possible explanation could be that these tumors are undergoing less glycolysis, creating an excess supply of glucose for infiltrating T cells (Harel et al., 2019). In addition, these tumors would also secrete less lactate, creating a more favorable environment for T cell-mediated killing (Harel et al., 2019). Overall, T cells face fierce competition for nutrients and are exposed to a multitude of toxic byproducts that can impair their function in the TME.

#### Extracellular vesicles

In addition to the concentration of metabolites, gradients in molecular elements like O_2_, and changes in physical characteristics of the TME such as pH, there are other discrete transferable factors that may also influence immune cell metabolism and function. Extracellular vesicles (EVs) encompass a diverse set of membrane vesicles secreted by most, if not all, cell types (Tkach et al., 2018). Tumors have been shown to secrete an abundance of EVs that can subsequently have biological effects on many different cell types, including immune cells (Chen et al., 2018; Ricklefs et al., 2018; Poggio et al., 2019). Under hypoxic conditions, pancreatic cancer cells secrete microRNAs into EVs that activate the PI3K signaling pathway to induce M2 macrophage polarization, which subsequently promotes cancer progression and predicts poor prognosis (Wang et al., 2018). Recent studies have begun to investigate metabolic effects of secreted EVs. In particular, both hepatic stellate cells and mutant KRAS colonic cells have been found to release EVs containing Glut1, which induces glycolysis in other cells in the TME (Wan et al., 2019; Zhang et al., 2018). In addition, proteomic and lipidomic analysis of EVs released from tumor associated macrophages were shown to contain a Th1/M1 signature and enzymes involved in lipid metabolism, which strongly correlated with an anti-tumor immune phenotype (Cianciaruso et al., 2019). The full range of effects of EVs on immune cell metabolism and function in anti-tumor immunity remains uncertain but has the potential to impact immunotherapy.

### Directly manipulating T cell metabolism to improve immunotherapy

There is considerable excitement surrounding ICB to treat cancer, which may derive its efficacy in part by altering T cell metabolism. Although both PD-1 and CTLA-4 impair utilization of glucose and glutamine by decreasing their uptake, these immune checkpoint proteins change lymphocyte metabolism through distinct molecular mechanisms (Parry et al., 2005; Patsoukis et al., 2015). In particular, PD-1 signaling blocks activation of PI3K and Akt in T lymphocytes, flipping a metabolic switch from glycolysis to lipolysis and fatty acid oxidation and thus impairing effector function (Patsoukis et al., 2015). A similar metabolic shift and decrease in cytokine production was observed upon PD-1 ligation activating STAT3 in CD8^+^ T cells, facilitating obesity-associated breast cancer progression (Zhang et al., 2020). These findings suggest that PD-1 blockade may function synergistically at two levels by re-invigorating T cell glycolysis while simultaneously inhibiting tumor cell glycolysis. On the other hand, binding of CD80 and CD86 to CTLA-4, a negative regulator of CD28 co-stimulation, inhibits glycolysis without affecting fatty acid oxidation, maintaining the metabolic program of quiescent cells and blocking this interaction can enhance CD28-mediated T cell metabolic reprogramming (Patsoukis et al., 2015). Like PD-1, ligand engagement of CTLA-4 also blocks Akt activation, but CTLA-4 performs this function in a PI3K-independent fashion (Parry et al., 2005). Thus, the suppressive effects of CTLA-4 on T cell activation may stem from its preservation of a bioenergetic profile similar to non-stimulated cells, while PD-L1 binding to PD-1 induces a distinct T cell metabolic state.

Modulating the metabolism of T cells presents an exciting avenue to improve current immunotherapies, in particular adoptive cell transfer, in which T cells are taken from a patient, primed, and expanded ex vivo before transfusing them back into the patient. This process requires careful manipulation of the T cells and presents an opportunity for discrete access to the cells for metabolic interventions. Several studies have shown that modulating the metabolism of adoptively transferred T cells with pharmacologic agents is a promising path to improve this form of immunotherapy (Chang and Pearce, 2016). Treating T cells in vitro during the priming and expansion phases with 2-deoxyglucose (2-DG), an inhibitor of glycolysis, increased memory T cell formation and subsequently enhanced antitumor function in vivo (Sukumar et al., 2013). Adoptive transfer of antigen-specific T cells treated ex vivo with an inhibitor of oxygen-sensing prolyl-hydroxylase domain proteins increased glycolytic activity and reduced lung metastasis in a B16-melanoma model (Clever et al., 2016). Similarly, treating T cells in vitro with Mdivi, an inhibitor of mitochondrial fission, also increases in vivo anti-tumor activity (Buck et al., 2016). Moreover, in vitro Akt pharmacologic inhibition increased the persistence of memory T cells after adoptive transfer and led to increased antitumor function in vivo (Crompton et al., 2015). Finally, adding bicarbonate to neutralize acidic environments improved response rates to checkpoint inhibition and adoptive cell therapy in mice models of melanoma (Pilon-Thomas et al., 2016). Taken together, these studies indicate that fine-tuning T cell metabolism in vitro prior to transfusion back into patients is key to their success in vivo.

### Conclusion

Disruption of T cell activation due to altered tumor cell metabolism and other metabolic features in the TME indicates that this is an important mechanism for immunosuppression. As tumors grow and proliferate, they rapidly consume nutrients such as oxygen and glucose and secrete lactate, creating regions of hypoxia and high acidity. Cancer cells and cells in the tumor microenvironment also shed EVs which may convey metabolic signals that can further hinder immune cell function. Upon entering the TME, TILs must overcome these metabolic challenges in order to mount a successful immune response. Although significant progress has been made in strengthening immunotherapy regimens, there remains significant knowledge gaps about how they work and why a majority of patients do not respond to treatment.

There are a number of challenges and opportunities to exploit the TME and immunometabolism in immunotherapy. One key challenge is to identify metabolic pathways that are cancer-specific or targets that can negatively influence cancers while improving the TME for immune function and not overly impairing immune cells. Targeting some pathways, such as glucose metabolism, may be challenging because both cancer and effector T cells and macrophages use and require these pathways. However, it was recently shown that other metabolic pathways, such as glutamine-dependent metabolism, may be more critical for cancer cells than inflammatory effector T cells or macrophages (Johnson et al., 2018; Leone et al., 2019; Liu et al., 2017), indicating that it may be possible to both target cancer metabolism and enhance immunity. The influence of tumor type, location, and diet on metabolites in the TME (Sullivan et al., 2019) though could necessitate context-specific interventions. Nevertheless, dietary modifications that affect nutrient availability in the TME and have shown promise in clinical trials to slow tumor growth, though context specific aspects may require distinct guidelines for different cancers (Kanarek et al., 2020).

Another instance where immunometabolism may be exploited to enhance immunotherapy is through adoptive cell therapy. Indeed, the potential to enhance the metabolic capacity of Chimeric Antigen Receptor (CAR)-T cells or other cells through in vitro manipulation prior to cell transfer may overcome barriers of unintended direct effects on the tumor cells. The potential side effects of metabolic modulatory drugs have also not been fully explored and must be considered as normal tissues may be affected. Nevertheless, metabolic inhibitors have had fewer toxicities than expected, possibly due to the lower metabolic activity and high degree of metabolic flexibility of most tissues. Key remaining questions include: Can we translate findings from experimental models to humans? How do we keep T cells alive long enough to form memory cells to reject future cancer cells? How do we balance activating anti-tumor T cells with autoimmune side effects or inadvertently enhancing cancer cell growth? Understanding the T cell metabolic program and how it underlies function and dysfunction represents a promising venue that can be exploited to improve immunotherapy efficacy.

## Disclosure statement

The authors declare no direct conflict of interest with the contents of this manuscript. JCR has hold stock equity in Sitryx and within the past two years has received unrelated research support, travel, and honorarium from Incyte, Sitryx, Caribou, Kadmon, Calithera, Tempest, Merck, and Pfizer. Within the past two years, WKR has received unrelated clinical research support Bristol-Meyers Squib, Merck, Pfizer, Peloton, Calithera, and Incyte. The content is solely the responsibility of the authors and does not necessarily represent the official views of the National Institutes of Health.
